# Epiretinal membrane surgery outcome in eyes with abnormalities of the central bouquet

**DOI:** 10.1186/s40942-020-00279-0

**Published:** 2021-01-14

**Authors:** Max P. Brinkmann, Stephan Michels, Carolin Brinkmann, Felix Rommel, Mahdy Ranjbar, Nicole Graf Johansen, Matthias Becker

**Affiliations:** 1 Department of Ophthalmology, Stadtspital Waid und Triemli Zürich, Zurich, Switzerland; 2 Department of Ophthalmology, University of Zürich, Zurich, Switzerland; 3 Laboratory for Angiogenesis & Ocular Cell Transplantation, University of Lübeck, Lübeck, Germany; 4 Graf Biostatistics, Winterthur, Switzerland; 5 Department of Ophthalmology, University of Heidelberg, Heidelberg, Germany; 6 Augenklinik Zürich West, Zurich, Switzerland

**Keywords:** Acquired vitelliform lesion, Epiretinal membrane, Foveal bouquet, Membrane peeling, Predictive factor, Vitrectomy

## Abstract

**Background:**

Clinical studies have shown that epiretinal membranes (ERM) as well as abnormalities of the central foveal bouquet (CB) can be classified in different stages according to their morphological appearance. Furthermore, visual acuity correlates with the different stages of these features. The present study evaluated how these findings change after the surgical removal of the ERM and their impact on functional outcomes.

**Methods:**

In this retrospective study eyes with ERM were evaluated by SD-OCT scans before and after pars plana vitrectomy (PPV) with macular ERM and internal limiting membrane (ILM) peeling. CB abnormalities were classified according to their morphological appearance from stage 0 (no abnormalities) to stage 3 (acquired vitelliform lesion). ERMs were classified ranging from stage 0 (absence of ERM) to stage 4 (ERM with significant anatomic disruption of macula). Changes in morphology were correlated with visual acuity before and after surgery.

**Results:**

151 eyes were included into the study. Before surgery 27.2% (n = 41) of eyes showed CB abnormalities with stage 1 being the most common (11.9%, n = 18). Before surgery ERM was seen in all patients. The most common form was stage 1 (28.5%, n = 43), followed by stage 3 (27.8%, n = 42) and 2 (25.2%, n = 38). Only 18.5% (n = 28) presented with stage 4 ERM. The mean BCVA was 0.42 (logMAR) before and increased to 0.19 (logMAR) 8 weeks after vitrectomy (95% CI 0.20–0.28; p < 0.001). Patients who suffered from CB abnormalities had less increase in BCVA than patients who had no evidence of CB (0.28 vs. 0.14 logMAR; p < 0.001). Of all the patients with CB abnormalities at baseline, 68% had lower CB grading after the surgery (n = 28; 95% CI; p < 0.001). All patients showed an improvement of their ERM grading, with 98.7% reaching stage 0 (n = 151 vs. n = 149; 95% CI; p < 0.001).

**Conclusions:**

The study indicates that the presence of CB abnormalities correlates with worse visual function. They are furthermore associated with worse visual outcomes after PPV with ERM and ILM peeling. These findings are valuable for deciding on PPV in patients with ERM.

## Background

Epiretinal membrane (ERM) formation is a common pathology of the retina with a prevalence ranging from 2.2 to 28.9% [1–3]. It is characterized by fibrocellular proliferations at the vitreoretinal interface, above the internal limiting membrane (ILM), which can cover the fovea partly or in total [4]. Most ERMs are idiopathic but they can also be associated with trauma, inflammatory disease, intraocular surgery or retinal detachment [5]. ERMs frequently result in reduced visual acuity and metamorphopsia [6]. The exact mechanism by which these impairments are caused is not yet fully understood. It is thought that tractional stress caused by the ERMs can induce changes such as increased retinal thickness, the formation of lamellar and full-thickness macular defects, or alterations of the outer foveal region [4, 7]. Additionally other cellular and vascular changes such as reduced uveal-scleral outflow, hemodynamic changes in choroidal flow, breakdown of the retinal pigment epithelium and disruption and leakage from the retinal capillary system have been discussed [7].

With high-resolution spectral-domain coherence tomography (SD-OCT) the possibility to investigate such changes and defining new descriptive terms has improved our pathophysiological understanding significantly [1, 8].

The central bouquet (CB) is a small circular island, less than 100 µm in diameter and located centrally at the fovea. In a recently published study, Govetto at al. investigated tractional abnormalities of the CB and furthermore postulated that these changes might be categorized into progressive stages beginning with the *cotton ball sign* followed by *foveolar detachment* and resulting in an *acquired vitelliform lesion* as the final stage [7, 9–12]. They also described a correlation between the anatomic progression and the corresponding best corrected visual acuity (BCVA). Due to these mechanisms, a progression in the anatomic stage is associated with a decline in BCVA in most cases [7].

Govetto et al. also developed a staging system for describing ERMs. In this system, a stage 1 ERM is only mild with negligible morphologic or anatomic disruption. At stage 2, a more progressive retinal distortion with loss of the foveal depression can be seen. Stage 3 is defined as an ERM with continuous ectopic inner foveal layers (EIFL) anomalously crossing the central foveal area, also with loss of the foveal depression. Finally, in stage 4 a significant retinal thickening and remarkable anatomic disruption of the macula is shown [4]. It was observed that more severe stages of ERM correlate with higher reduction of visual acuity [4].

Pars plana vitrectomy (PPV) with epiretinal membrane and internal limiting membrane (ILM) peeling is the standard surgical approach to release tension and restore the normal structure of the retina [13–20]. However, despite high anatomical success rates, the postoperative visual outcome can be most variable despite surgical success [20–23].

The aim of this study is to evaluate the impact of different stages of ERM and CB abnormalities on the functional and anatomic outcomes following PPV with ERF and ILM peeling.

## Methods

**Fig. 1:**
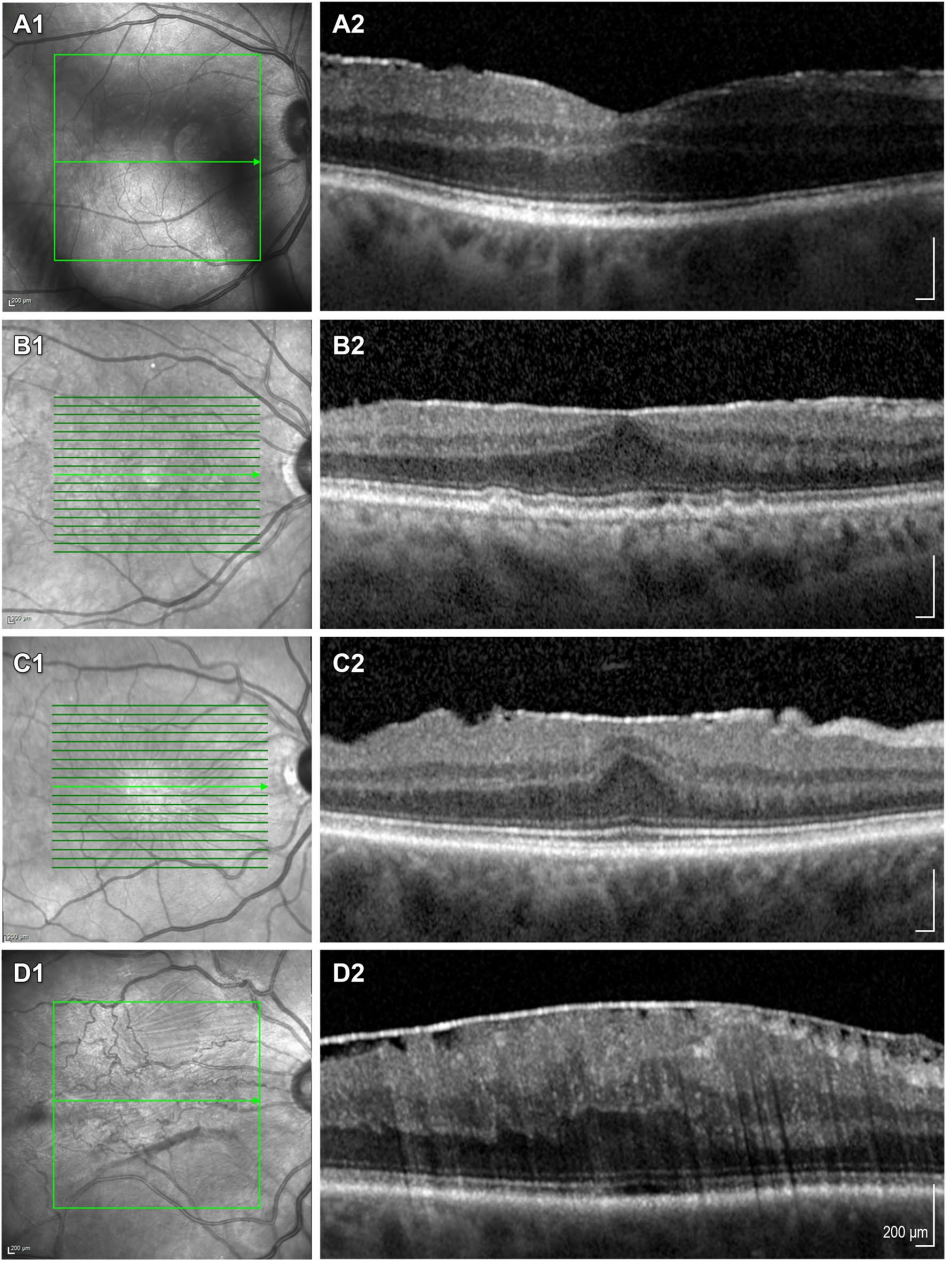
Staging scheme for epiretinal membranes. **a1–c1** Show near-infrared images, **a2**–**c2** display the corresponding SD-OCT scans of ERM stage as proposed by Govetto et al. [4]: **a1**, **a2** stage (1) Mild ERM with few anatomical modifications. The foveal depression is preserved, and all retinal layers are well identified. **b1**, **b2** stage (2) ERM with more advanced anatomical changes. The foveal depression is lost, but all retinal layers are still well defined. **c1**, **c2** stage (3) Continuous ectopic inner foveal layers (EIFL) cover the whole foveal floor. Like in Stage 2 ERMs, the foveal depression is lost, and all retinal layers are well defined. **d1**, **d2** stage (4) Advanced ERM with complete foveal disorganization. Thick EIFLs cover the foveal area, there is no foveal depression, and all retinal layers are disrupted [27]

**Fig. 2:**
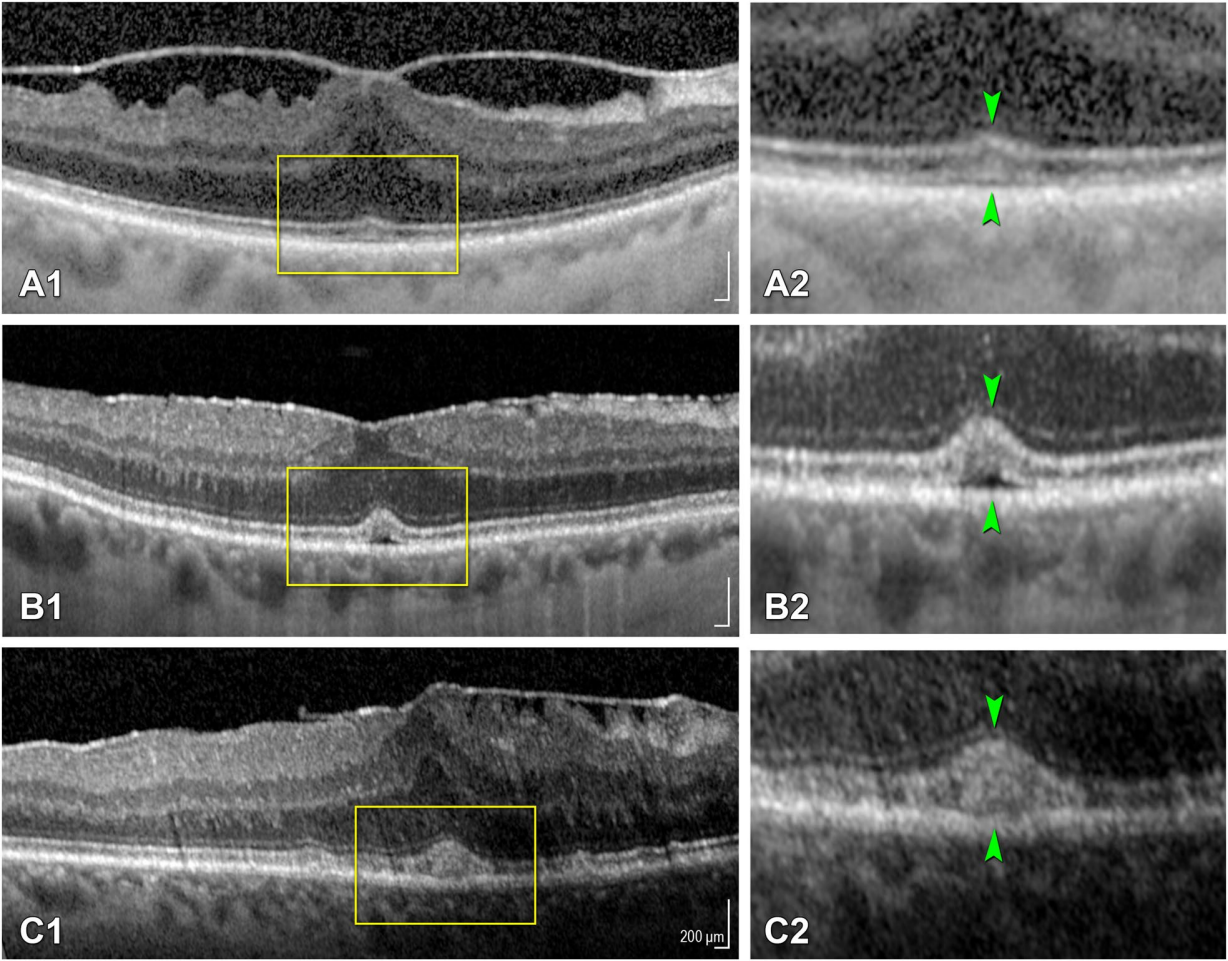
Tractional abnormalities of the central bouquet. **a1**–**c1** The corresponding magnifications **a2**–**c2** show SD-OCT scans through the foveal region representing the different stages of CB abnormalities as proposed by Govetto et al. [7]: **a1**, **a2** stage (1) A small, fuzzy hyperreflective area (*cotton ball sign*, green arrowheads). **b1**, **b2** stage (2) A central hyporeflective pocket of subretinal fluid under the interdigitation zone (green arrowheads). **c1**, **c3** stage (3) A thick dome-shaped hyperreflective acquired vitelliform lesion between the retinal pigment epithelium and the ellipsoid zone (green arrowheads) [7]

In a retrospective, consecutive study we evaluated patients suffering from ERM and having received surgical treatment with 23 g or 27 g pars plana vitrectomy and ERM and ILM peeling at the Triemli City Hospital Zurich between 2014 and 2018. The study was approved by the local ethics committee. Eyes with other diseases potentially affecting surgical outcomes (e.g. age-related macular degeneration, diabetic maculopathy, retinal vein occlusions) were excluded. All patients underwent a 23 or 27 three port vitrectomy with ERM peeling, staining of the ILM with Membrane Blue Dual (DORC, Netherlands) and ILM peeling by two vitreoretinal surgeons (SM and MB). All patients received a complete ophthalmologic assessment, including slit-lamp biomicroscopy and dilated fundus examination. For statistical analysis both prior to and 6–8 weeks following surgery, Snellen visual acuity was measured and converted into the logarithm of the minimum angle of resolution (logMar). All eyes were evaluated by Heidelberg Spectralis Spectral Domain OCT System (SD-OCT) (Heidelberg Engineering, Heidelberg, Germany) and were classified prior to surgery and 8 weeks after surgery according to their morphological appearance. We used the classification systems of Govetto et al. for describing stages of ERM (Fig. 1) and alterations of the central bouquet (Fig. 2).

### Statistical analysis

**Fig. 3:**
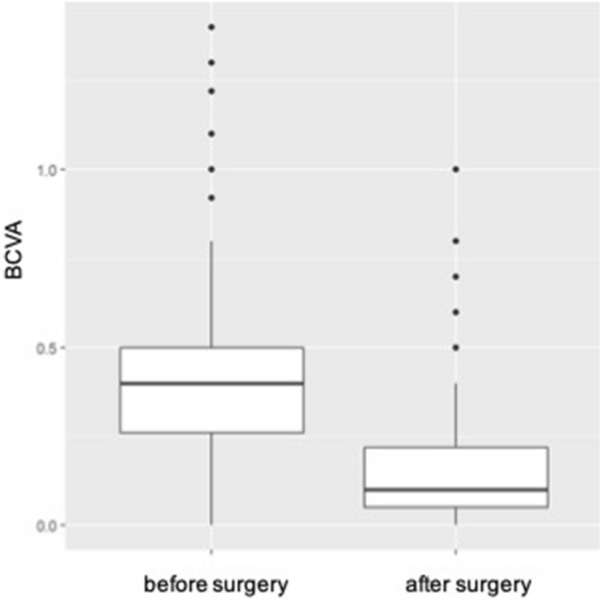
Boxplot of BCVA before and after surgery. The line in the box shows the median, the lower and upper hinges correspond to the first and third quartiles, the upper/lower whisker extends from the hinge to the largest/smallest value no further than 1.5 * IQR from the hinge

**Fig. 4:**
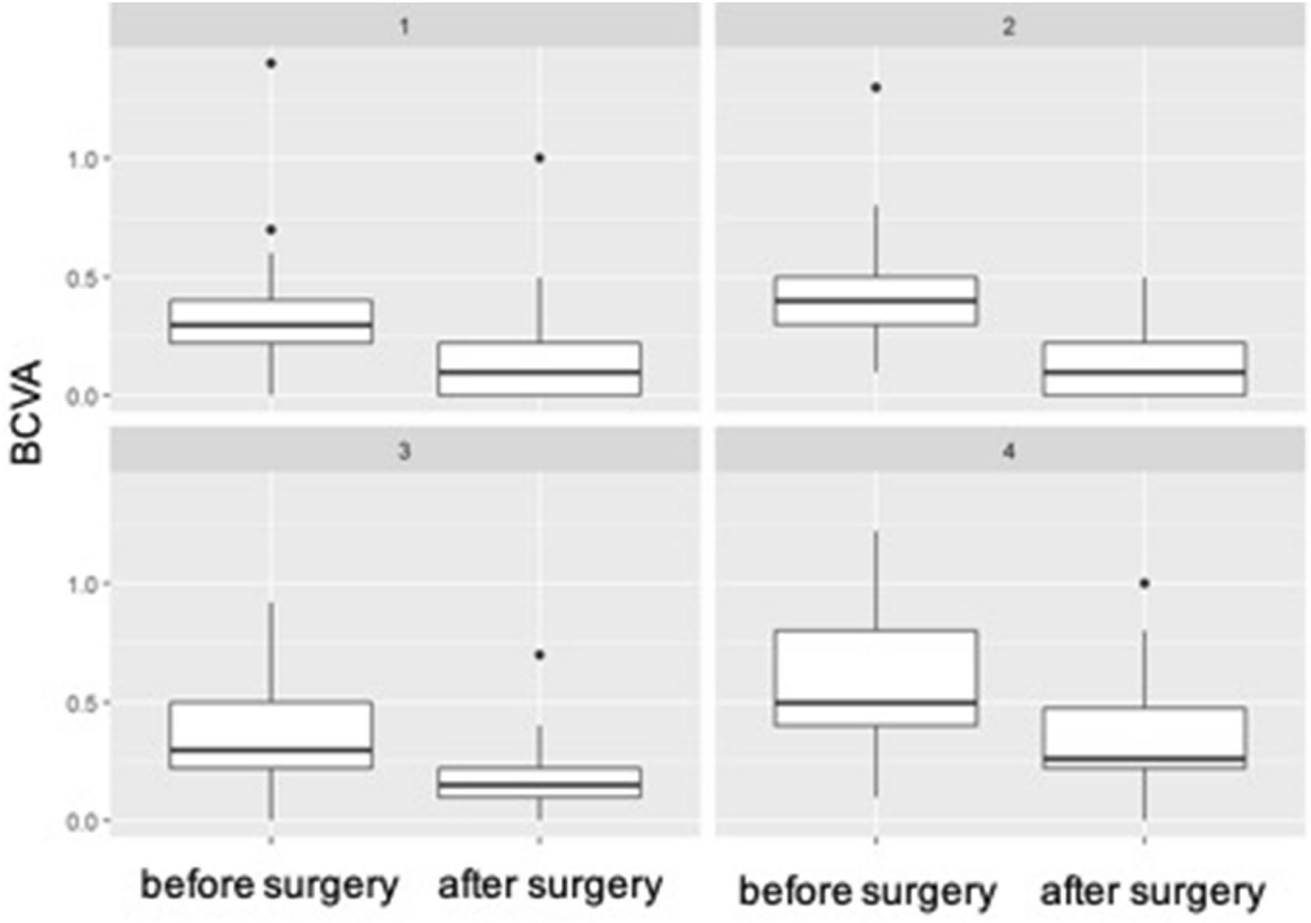
Boxplot of BCVA before and after surgery for baseline ERM (stages 1–4). The line in the box shows the median, the lower and upper hinges correspond to the first and third quartiles, the upper/lower whisker extends from the hinge to the largest/smallest value no further than 1.5 * IQR from the hinge

Ordinal variables (Alterations of the central bouquet, ERM) were compared with an exact sign test. Visual acuity was compared with a dependent-samples t-test as the differences between pre- and postoperative findings were distributed normally. Patients with no alterations of the CB were compared to those showing type 1–3 CB abnormalities with a Wilcoxon rank-sum test for changes in vision. Two-sided tests were performed and p-values < 0.05 were considered significant. Alpha was not adapted for multiple testing. All analyses were performed in the R programming language (version 3.3.3) (R Core Team, 2017) (Figs. 3, 4).

## Results

151 patients were included in the retrospective study. 47 (31%) were female, 104 (69%) were male. Preoperative vision was 20/50 and ranged from 20/500 to 20/20. The mean age was 74 years. In 92 (61%) cases surgeries were combined with clear cornea cataract surgery in the same session. Patients were seen on a mean of 6.8 weeks after surgery. Prior to surgery 27.2% (n = 41) of the patients showed foveal changes with stage 1 being the most common (15.9%, n = 24) (Table 1). 58% of eyes showing alterations of the central foveal bouquet were classified as stage 1, 20% as stage 2 and 22% as stage 3. Of all the patients with foveal changes, 68% had lower grading of CB alteration after the surgery than before (n = 28; 95% CI; p < 0.001). Only 3.3% of patients showed an increase in their CB alteration stage (n = 5). For the nine patients showing the most severe form of CB abnormality (stage 3), three patients did not show an improvement in their stage. One patient was classified as stage 2 after the surgery, three as stage 1. Two patients did not show any changes of the central foveal bouquet after the procedure. Half the eyes demonstrating stage 2 alterations before intervention were graded as stage 0 afterwards. Most patients with originally stage 1 showed no signs of CB alteration after surgery was performed. In one case, a stage 1 alteration developed into stage (2) Four patients who in the beginning had no signs of altered CB showed grade 1 alteration at the follow-up OCT. All eyes (n = 151) had ERM before surgery with stage 1 being the most common classification (28.5%, n = 43) (Table 1) and all eyes showed an improvement of their ERM grading, with 98.7% reaching stage 0 (n = 151 vs. n = 149; 95% CI; p < 0.001).

**Table 1:** Distribution of the different stages of alterations of the central bouquet and epiretinal membranes before and after surgery

Stages of CB alteration	0 (no CB alteration)	1	2	3	
Baseline	110 (72.8%)	24 (15.9%)	8 (5.3%)	9 (6.0%)	
End of follow-up	127 (84.1%)	18 (11.9%)	3 (2.0%)	3 (2.0%)	

Stages of ERM	0 (no ERM)	1	2	3	4
Baseline	0	43 (28.5%)	38 (25.2%)	42 (27.8%)	28 (18.5%)
End of follow-up	149 (98.7%)	1 (0.7%)	0	1 (0.7%)	0

Top row: Distribution of CB alteration stages at baseline and after surgery. Bottom row: Distribution of ERM stages at baseline and after surgery. Remaining ERMs fragments were detected in the OCT follow-up exam after surgery in only two patients. In both cases the ERM stage was worse before than after the procedure. The mean BCVA was 20/50 (0.42 logMAR) before and 20/32 (0.19 logMAR) after the procedure indicating a significant mean gain in vision of almost 2.5 lines (95% CI 0.20–0.28; p < 0.001) (Fig. 3). No significant difference in BCVA was observed comparing surgeries including cataract removal to those without (p = 0.349). Patients who showed foveal changes prior to surgery had less BCVA increase than patients classified as CBA 0 (0.28 vs. 0.14 logMAR; p = (p < 0.001) (Table 2). Looking at the different stages of ERMs before surgery, vision gain was distributed almost equally between the stages, reaching no statistical significance. However, greatest improvement was reached by stage 4 ERM eyes after removal of the membrane (mean vision gain = 0.28), the smallest by ERM stage 1 eyes (mean vision gain = 0.2) (Fig. 4)

*CB* central bouquet, *ERM* epiretinal membrane

**Table 2:** Changes of BCVA before and after surgery, stratified into different groups

Total number of patients (n)	151			
BCVA (logMAR) (mean (sd))	Before surgery	After surgery	Difference	
	0.42 (0.24)	0.19 (0.19)	− 0.24 (0.23)	
Stages of CB abnormalities	Not present	1	2	3
n	110	24	8	9
Difference BCVA (logMAR) before/after surgery (mean (sd))	− 0.28 (0.24)	− 0.14 (0.15)	− 0.08 (0.20)	− 0.18 (0.12)
Presence/absence of CB abnormality	Present	Not present		
n	110	41		
Difference BCVA (logMAR) before/after surgery (mean (sd))	− 0.28 (0.24)	− 0.14 (0.15)		
Stages of ERM before surgery	1	2	3	4
n	43	38	42	28
Difference BCVA (logMAR) before/after surgery (mean (sd))	− 0.20 (0.29)	− 0.27 (0.21)	− 0.21 (0.19)	− 0.28 (0.21)

Top row: all eyes before vs. after surgery; 2nd row from top: stratified into different stages of CB abnormalities; 3rd row from top: all eyes showing CB abnormalities vs. those without; bottom row: stratified into different ERM stages prior to surgery)

*BCVA* best corrected visual acuity, *CB* central bouquet, *logMAR* logarithm of the minimum angle of resolution

**Fig. 5:**
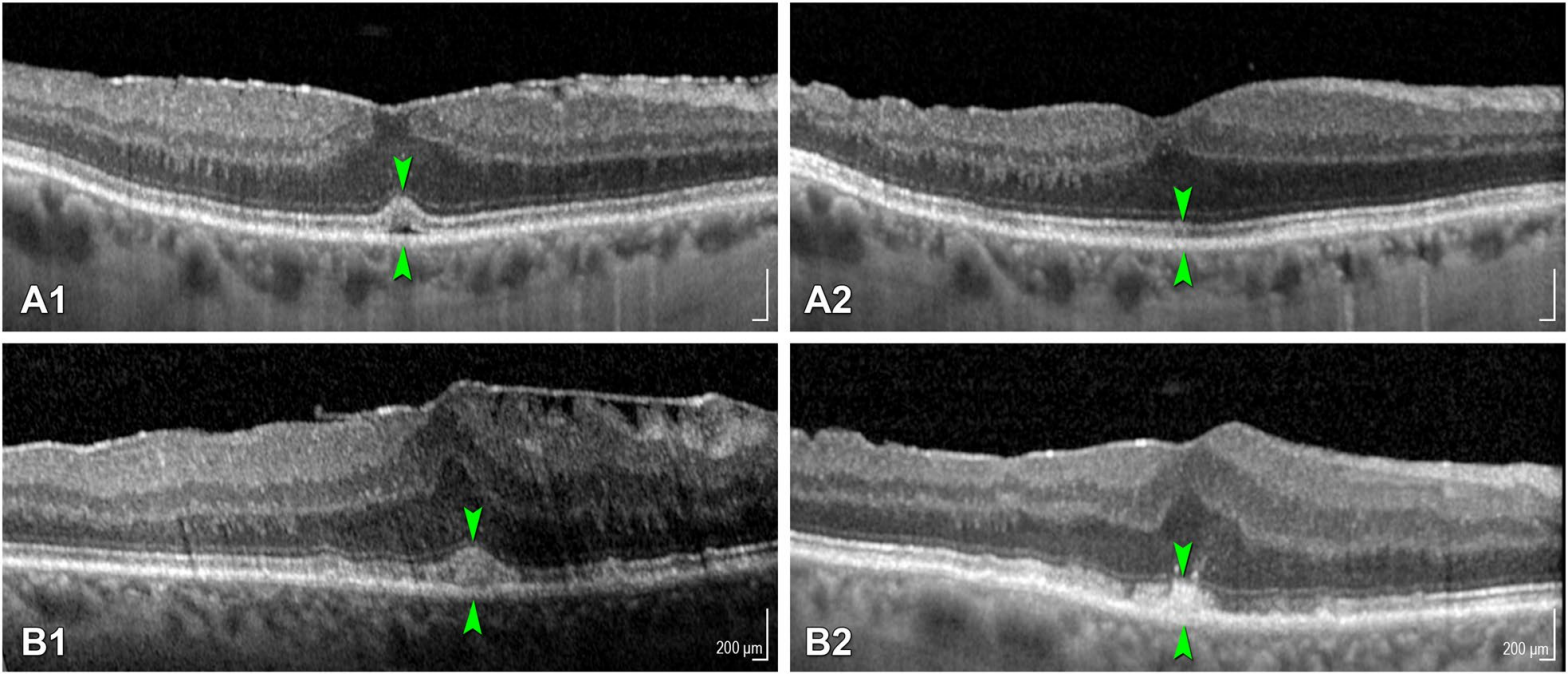
Optical coherence tomography imaging of two patients showing abnormalities of the central bouquet. **a1**, **a2** SD-OCT images 10 weeks before and 7 weeks after ERM removal. **a1** Before the procedure a hyporeflective area between the RPE and the external limiting membrane (ELM) could be identified (between green arrowheads), representing a type 2 CB abnormality (subretinal pouch), associated with a type 2 ERM. **a2** 7 weeks after ERM removal, a physiological configuration of the outer retina was observed. **b1**, **b2** display the change of a type 3 CB abnormality before and after membrane peeling. **b1** 5 weeks before surgery, a hyperreflective subretinal mass (between green arrowheads) was associated with a type 4 ERM. **b2** 6 weeks after ERM removal retinal thickness decreased from 550 to 480 µm, the subretinal material appeared less solid with outlines disappearing

Figure 5 displays OCT imaging before and after surgery of two patients showing great morphological improvement.

## Discussion

The goal of this study was to evaluate the impact of alterations of the central foveal bouquet and stages of epiretinal membranes on functional and anatomic changes after pars plana vitrectomy with ERM and ILM peeling. The central foveal region was assessed using Spectral Domain OCT allowing the detection of alterations of the central bouquet and epiretinal membrane stages. Besides the influence on visual acuity by the performed surgery, the impact of surgery on the configuration of the central foveal region was analyzed.

A significant overall mean gain in BCVA of 2.5 lines was achieved and ERM was completely peeled in the central macular region in 98.7%. The presence of alterations of the CB was a clear indicator for poorer functional outcome in short term follow-up, however the majority of eyes (68%) showed an improvement in their foveal changes at 6–8 weeks. Further follow-up is clearly needed to evaluate the long-term anatomic and functional evolution. The stage of the pre surgery ERM did not have a statistically significant impact on the mean change in BCVA at 6–8 weeks, indicating that all stages benefit from surgery, however higher levels of visual acuity can be preserved when surgery is performed at an earlier stage. To our knowledge other studies have already described modifications of the outer retinal morphology [10, 11], while the influence of surgical removal of epiretinal membranes on specific changes in the CB and the associated functional outcome has not yet been investigated. In our cohort the prevalence of alterations of the central foveal bouquet as well as the distribution of the different types of these changes was comparable to that of previous studies [7, 24]. Before surgery 27.2% (n = 41) of the patients showed subfoveal changes with stage 1 being the most common (15.9%, n = 24) (Table 1) [7]. Like Govetto et al. our findings confirm a correlation between BCVA and the stages of ERMs. The higher the ERM stage prior to surgery, the lower the visual acuity was [4, 25].

As expected most of the epiretinal membranes could be removed completely in the central foveal region (98.7% reaching grade 0, n = 151 vs. n = 149; 95% CI; p < 0.001) leading to a significant gain in vision of almost 2.5 lines (95% CI 0.20–0.28; p < 0.001) [26].

Examination of the anatomical changes showed that 68% of the patients with changes of CB, had lower grading after the surgery (n = 28; 95% CI; p < 0.001). In addition, patients who presented with foveal changes prior to surgery had less BCVA increase than patients without (0.28 vs. 0.14 logMAR; p = (p < 0.001)).

Comparing the procedures that were including cataract removal to those without, no significant difference could be observed in the in the visual and morphological outcome.

Disadvantages of our study are clearly the retrospective nature and the relatively short follow-up in a rather large cohort.

## Conclusions

The results of this study clearly state that foveal changes secondary to ERM are relevant and an important independent negative predictor for functional outcomes following PPV with ERM and ILM peeling for ERM. Therefore, precise evaluation of the fovea and classification of possible CB abnormalities appears a valuable tool in pre-surgical evaluation, potentially even more valuable than the stage of ERM.

## Data Availability

The datasets used and/or analysed during the current study are available from the corresponding author on reasonable request.

## Abbreviations

BCVA: Best corrected visual acuity
CB: Central foveal bouquet
ERM: Epiretinal membrane
ILM: Internal limiting membrane
PPV: Pars plana vitrectomy
SD-OCT: Spectral-domain coherence tomography
